# Data detectives, self-love, and humility: a research parasite's perspective

**DOI:** 10.1093/gigascience/giz148

**Published:** 2020-01-03

**Authors:** Claire Duvallet

**Affiliations:** Biobot Analytics, 444 Somerville Avenue, Somerville, MA 02143, USA

## Abstract

Secondary analysis solidifies and expands upon scientific knowledge through the re-analysis of existing datasets. However, researchers performing secondary analyses must develop specific skills to be successful and can benefit from adopting some computational best practices. Recognizing this work is also key to building and supporting a community of researchers who contribute to the scientific ecosystem through secondary analyses. The Research Parasite Awards are one such avenue, celebrating outstanding contributions to the rigorous secondary analysis of data. As the recipient of a 2019 Junior Research Parasite Award, I was asked to provide some perspectives on life as a research parasite, which I share in this commentary.

## Introduction

In sharp contrast with some unfounded fears that open data will lead to the rise of “research parasites” [1], publicly available raw data adds substantial value to scientific research, allowing researchers to ask and answer new questions and to test hypotheses *in silico* without the need for costly new studies. In my PhD work, I performed a meta-analysis of 28 gut microbiome studies, where I downloaded and reprocessed raw sequencing data to look at patterns of associations between the gut microbiome and 10 different diseases [2]. When I first embarked on this project, I thought it would be straightforward to use existing data to answer some low-hanging-fruit questions in my field. But I soon learned that performing a meta-analysis is more than a “stamp collection” endeavor and is rather a unique, difficult, and valuable contribution to the field [3]. As the recipient of the 2019 Junior Research Parasite Award, I was recognized for my contribution to the rigorous secondary analysis of microbiome data and given this opportunity to share some of the lessons I learned [4].

**Figure 1:**
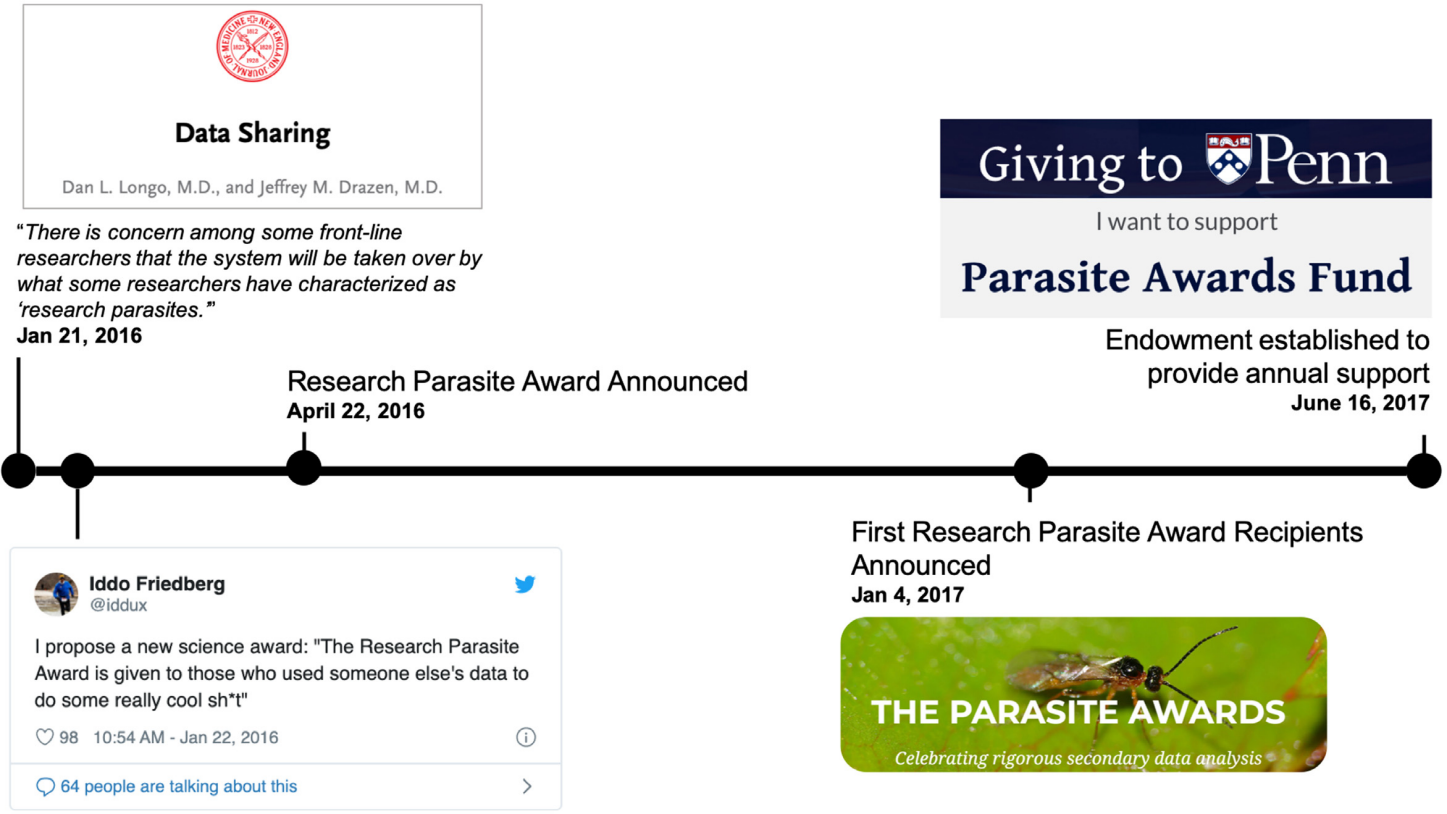
Timeline of the creation of the Research Parasite Award, which recognizes exemplary data reuse, considering in particular that the ideal parasite does not kill its host.

## Research Parasites Are Data Detectives

First, a research parasite must become a data detective. While databases and search engines ease the process of finding data, identifying a comprehensive list of datasets for niche research questions remains challenging. In my meta-analysis, I learned to supplement my database searches by chasing down references from papers and reviews. I became skilled at scrutinizing papers to find links to raw data and metadata, information that was often scattered throughout the paper and supplement. I eventually gained some intuition: if a ctrl-F for certain data-related keywords was not fruitful, I knew I should probably go ahead and email the authors. But even that required detective work in order to find up-to-date email addresses and, at times, contact information for the first author, who might be more inclined to reply to my email. Through every step of the process, I learned to notice strategic details and scour available information for any clues that might help me find what I needed.

## Data Without Metadata Is Useless

Without all of the relevant technical and clinical metadata, it is impossible to re-analyze data and the entire dataset becomes useless. As a parasite, I identified 3 broad types of dataset-related metadata: (i) metadata linking the raw data IDs (e.g., file names, SRA run IDs) to their corresponding sample IDs, (ii) metadata containing technical processing information (e.g., sequencing barcodes, sample replicate number, sampling date), and (iii) biological or clinical metadata (e.g., disease status, tissue type). Here also, there are many tricks to learn, e.g., looking at raw sequencing data to infer the presence or absence of primers or barcodes, making educated guesses about disease status from sample IDs, and looking in all the nooks and crannies of SRA or ENA sample descriptors.

**Figure 2:**
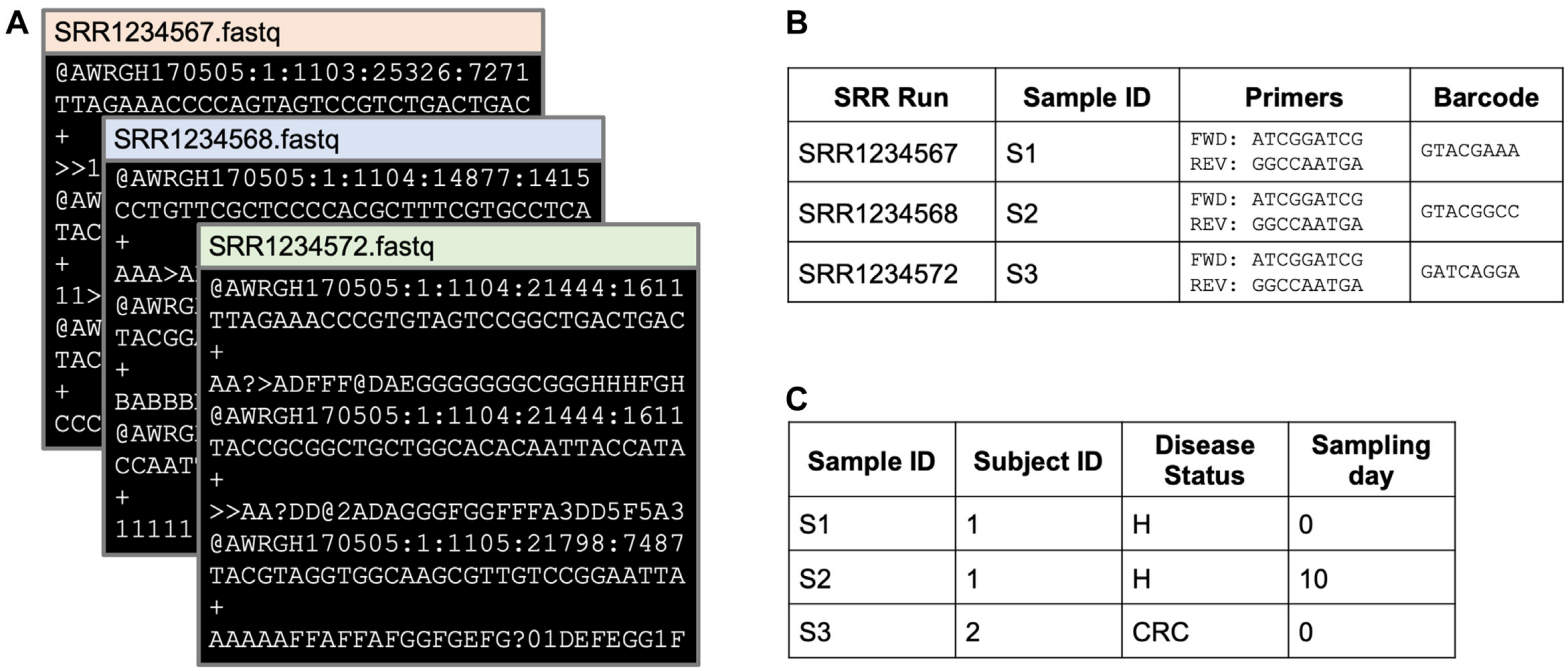
Data and metadata components required for a successful re-analysis. (A) Raw sequencing data are usually labeled with an SRR Run ID or other processing ID. Raw data rarely contain information about the processing steps applied, and the research parasite must use other information to determine what processing needs to be done. (B) Technical metadata connect file names to the respective sample ID and may also have other technical information such as primer and barcode sequences. In cases in which the raw sequencing data have not been separated into sample-wise files, barcodes are required to map sequences to samples. These are often the most difficult data to find. (C) Finally, study-related metadata are required to re-analyze samples. Metadata directly related to the analysis question are always necessary (i.e., disease status), but other metadata such as subject ID, sampling day, and replicate may also be required to ensure that proper statistical comparisons are being made.

## Organize Yourself for Success

As research parasites, we make mistakes and need to start analyses over from scratch many times. Intentional project and file organization can ensure that starting over is not a catastrophe each time. More specifically, separating source code from the data and producing intermediate files for each step of an analysis is key to unlocking parasitic productivity [5, 6]. Clear project organization makes it easier to remember what different files are, to implement automated pipeline workflows, and to write parallel and modular scripts.

**Figure 3:**
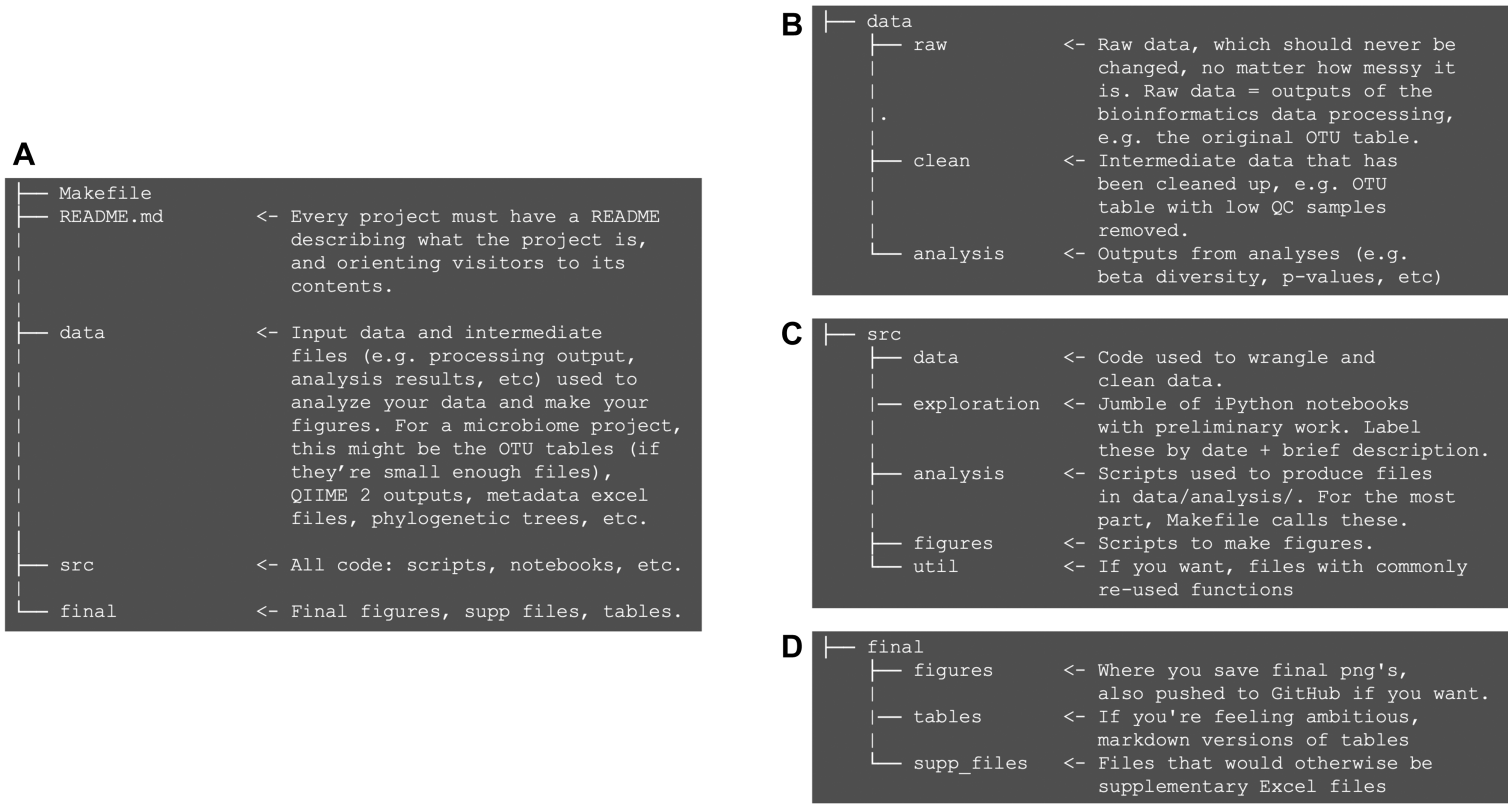
A sample project structure, adapted from Cookie Cutter Data Science [5] for my meta-analysis. In microbiome projects, operational taxonomic unit (OTU) tables are the feature tables that result from processing raw sequencing data, and that serve as the input to all analyses. (A) Overall project structure; (B, C, and D) individual folder structures. QC: quality control.

## Documentation Is Clues for Future-You

In a way, README files are a research parasite′s lab notebook. Re-analyzing other people's datasets often means long and convoluted paths from raw data to insight. It is important to document every step along this path: how the data were downloaded and processed, including any strange things that happened and why certain decisions were made; how the scripts and data are organized and where all the (raw) data live; and how others can use or build upon what has been done.

As a research parasite, the most likely beneficiary of thorough documentation is future-you, checking your work before submission, looking for code to do something you know you have already figured out, or going back to answer a question that woke you up in a cold sweat at 2:00 a.m. This was a lesson learned the hard way for me: I remember going back to finalize processing on datasets I had downloaded >1 year prior, and not finding any clues from myself to indicate what I had done. In all of these cases, it was faster to re-download the data from scratch than to time-travel into the past and remember what I had been thinking.

## The Life-Changing Magic of Makefiles

**Figure 4:**
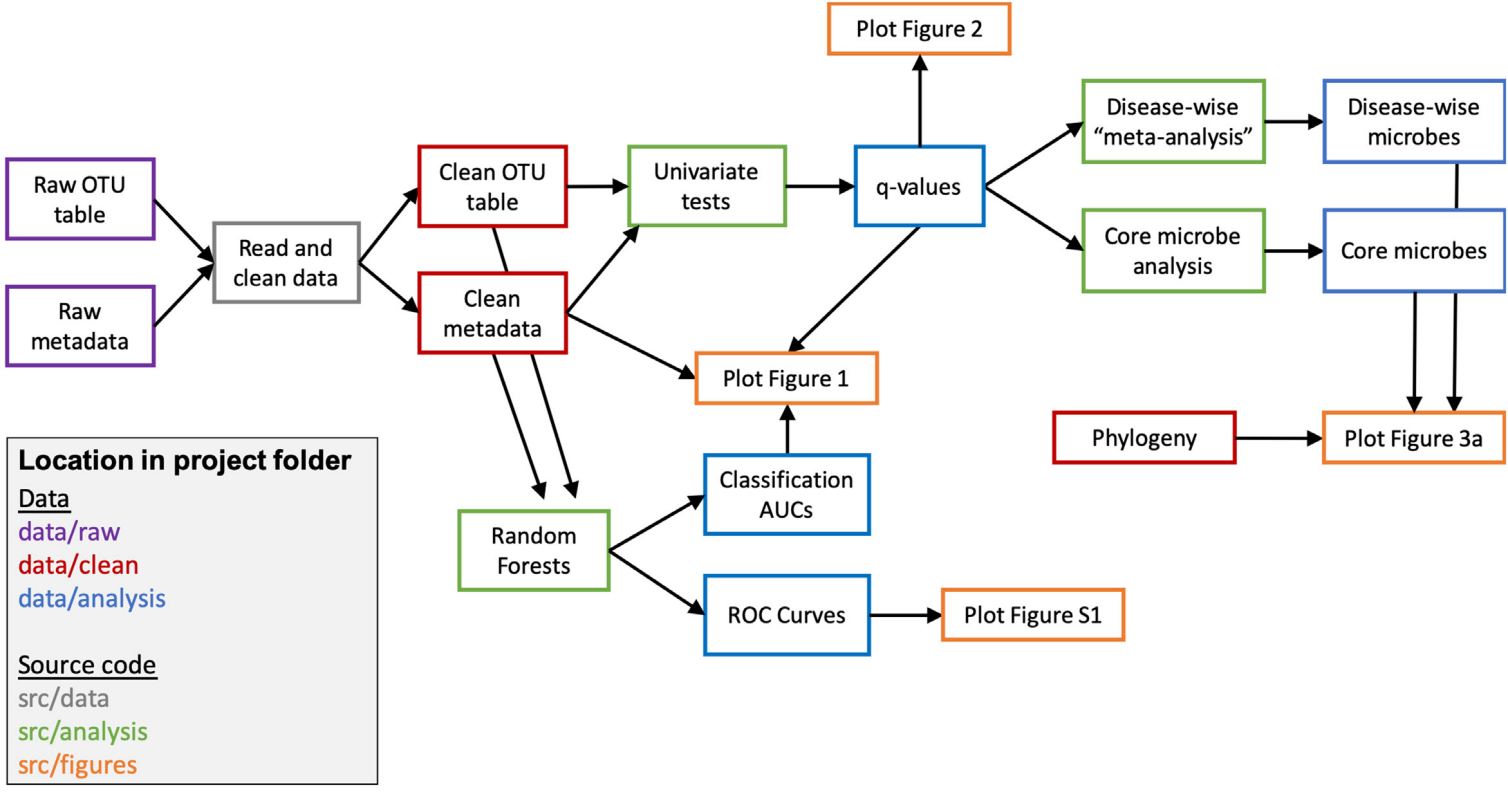
Schematic of a subset of the steps involved in the workflow for my meta-analysis. Each box contains a description of the data or analysis at that step, and arrows indicate progression through the analysis workflow. Box color indicates respective location in the project structure. Dependencies between input data, intermediate files, and scripts are encoded in the Makefile, which automatically reruns any necessary steps when input data or files are updated. For example, if the script to perform univariate steps is updated, all steps that depend on that box get rerun and updated; i.e., Figs 1, 2, and 3a are all remade. In contrast, if the code to run the random forests is updated, only Figs 1 and S1 are updated. AUC: area under the ROC curve; ROC: receiver operating characteristic.

In my opinion, the most revolutionary parasitic tactic of all is pipelining workflows with Makefiles. Broadly, Makefiles contain all of the instructions to reproduce every part of an analysis and are usually paired with a software program that reads the instructions in the Makefile to automatically rerun all parts of the analysis that need to be updated (Figure 4) [7, 8]. Makefiles are incredibly useful because they enable the remaking of an entire analysis and—more importantly—they are by definition a documentation of all the steps taken. They quite literally answer the question, “wait, how did I do that again?”

Makefiles also make the review process much easier, saving time and freeing research parasites from tedious time-consuming details. With a Makefile, it is easy to go back, find, and double-check individual steps in an entire analysis, as opposed to digging through folders of spaghetti code without guidance. Adding new analyses also becomes easier, as new scripts can be seamlessly plugged into the Makefile, using intermediate analysis files already created from other steps in the workflow. And most importantly, Makefiles take care of the details, automatically updating figures and tables when their underlying inputs change, saving research parasites many hours of tedious detail-checking.

## Computational Best Practices as an Act of Radical Self-Love

Documentation and pipelining are two manifestations of an important philosophy that is crucial to the research parasite′s success: implementing best practices in computational research is an act of radical self-love. Acting in self-love means approaching all decisions not for current-you, but instead for the person who will bear the consequences of your decisions: future-you, long after you have forgotten anything you thought today. It is not possible to enumerate all of the possible best practices or even to identify which ones are practical to implement as PhD students, but I found that as long as I made decisions for future-me rather than only current-me, my life as a parasite drastically improved.

## Democratized Software Catalyzes Data Generation by Non-specialists

Throughout my PhD work, I realized that being a parasite is not just about data: developing software tools that encourage non-bioinformaticians to analyze their own datasets is also key to a future where secondary analyses are the norm. In the microbiome field, we have seen a recent development of incredibly accessible bioinformatics tools that democratize access to computational analyses [9]. I hypothesize that these easy-to-use analysis tools have contributed to an increase in non-specialists generating microbiome sequencing data.

For example, many microbiome-related clinical trials now include microbiome analyses as secondary end points or to raise the impact of a study. These analyses would be inaccessible without the easy-to-use software being developed by the computational microbiome community. Interestingly, these studies rarely dive into the full potential of their data because they are focused on their primary clinical end points. That leaves a lot of room for research parasites to come in and ask different questions of those datasets, which I think is a great by-product of developing accessible software suites.

## Practice Humility and Broaden Your Perspective

Finally, the biggest lesson I learned from my life as a parasitic PhD student was humility. As a research parasite, I found it easy to get self-righteous and angry whenever the data or metadata are not easily accessible, but at the end of the day it is not productive. Especially as junior PhD students, we must recognize that what we view as just additional N's in our analyses, clinicians see as real people who gave their time and samples to advance research, and in some cases, as patients who can be deeply suffering.

Humility and empathy are also important to recognize that while we stand on our soapboxes advocating for open and reproducible science, it is actually really difficult to do well. An example is data sharing: parasites might like to grumble at how hard it is to find and download raw data, but depositing data is itself a complicated process. Data generation is a collaborative, lengthy process, and the person depositing the data is rarely involved in every step of the data generation. Even after downloading and processing these 28 datasets, I myself was incredibly confused when I needed to deposit data for a different project that I worked on, and I am sure I missed some important information that will make a future parasite grumble herself. Thus, not only do we need to require data sharing, but we also need to make it easier, more accessible, and more amenable to improvements and feedback, in addition to encouraging and rewarding those who do share their data (e.g., like the Research Symbiont Award [10], a partner to the Research Parasite Award). And as we are doing our parasitic research, we need to keep in mind that we are just one small part of an entire ecosystem, and that as parasites, we depend on our hosts to survive.

## Note from the Editors

The 2020 Research Parasite Award will again be held at the Pacific Symposium on Biocomputing and will be presented on 5 January 2020 at the Fairmont Orchid on the Big Island of Hawaii. As promoters of data sharing *GigaScience* has each year sponsored the Junior Parasite Award for postdoctoral, graduate, or undergraduate trainees and is again proud to support the award with a travel grant. For more see the Research Parasite Awards website, https://researchparasite.com/.

## Abbreviations

AUC: area under the curve; ENA: European Nucleotide Archive; ROC: receiver operating characteristic; SRA: Sequence Read Archive.

## Competing Interests

The author declares that she has no competing interests.
